# On the Asymptotic Optimality of a Low-Complexity Coding Strategy for WSS, MA, and AR Vector Sources

**DOI:** 10.3390/e22121378

**Published:** 2020-12-05

**Authors:** Jesús Gutiérrez-Gutiérrez, Marta Zárraga-Rodríguez, Xabier Insausti

**Affiliations:** Tecnun, University of Navarra, Paseo Manuel Lardizábal 13, 20018 San Sebastián, Spain; mzarraga@tecnun.es (M.Z.-R.); xinsausti@tecnun.es (X.I.)

**Keywords:** source coding, low-complexity, wide sense stationary (WSS) vector source, moving average (MA) vector source, autoregressive (AR) vector source

## Abstract

In this paper, we study the asymptotic optimality of a low-complexity coding strategy for Gaussian vector sources. Specifically, we study the convergence speed of the rate of such a coding strategy when it is used to encode the most relevant vector sources, namely wide sense stationary (WSS), moving average (MA), and autoregressive (AR) vector sources. We also study how the coding strategy considered performs when it is used to encode perturbed versions of those relevant sources. More precisely, we give a sufficient condition for such perturbed versions so that the convergence speed of the rate remains unaltered.

## 1. Introduction

In [1], Kolmogorov gave a formula for the rate distortion function (RDF) of Gaussian vectors and for the RDF of Gaussian wide sense stationary (WSS) sources. In [2], Pearl presented an upper bound for the RDF of finite-length data blocks of any Gaussian WSS source and proved that such a bound tends to the RDF of the source when the length of the data block grows. However, he did not propose a coding strategy to achieve his bound for a given block length. In [3], we presented a tighter upper bound for the RDF of finite-length data blocks of any Gaussian WSS source, and we proposed a low-complexity coding strategy to achieve our bound. Obviously, since such a bound is tighter than the one given by Pearl, it also tends to the RDF of the source when the length of the data block grows. In [4], we generalized our low-complexity coding strategy to encode (compress) finite-length data blocks of any Gaussian vector source. Moreover, in [4], we also gave a sufficient condition for the vector source in order to make such a coding strategy asymptotically optimal. We recall that a coding strategy is asymptotically optimal if its rate tends to the RDF of the source as the length of the data block grows. Such a sufficient condition requires the Gaussian vector source to be asymptotically WSS (AWSS). The definition of the AWSS process was first introduced in [5], Section 6, and extended to vector processes in [6], Definition 7.1. However, the convergence speed of the rate of the coding strategy considered (i.e., how fast the rate of the coding strategy tends to the RDF of the AWSS vector source) was not studied in [4].

In this paper, we present a less restrictive sufficient condition for the vector source to make the coding strategy considered asymptotically optimal. Moreover, we study the convergence speed of the rate of such a coding strategy when it is used to encode the most relevant vector sources, namely, WSS, moving average (MA), and autoregressive (AR) vector sources. In this paper, we also study how the coding strategy considered performs when it is used to encode perturbed versions of those relevant sources. Specifically, we give a sufficient condition for such perturbed versions so that the convergence speed of the rate remains unaltered.

The study of the convergence speed in any information-theoretic problem is not an easy task. To study the aforementioned convergence speed, we first need to derive new mathematical results on block Toeplitz matrices and new mathematical results on the correlation matrices of the WSS, MA, and AR vector processes. These new mathematical results are useful not only to study the convergence speed in the information-theoretic problem considered, but also in other problems. In fact, as an example, in Appendix H, we use such mathematical results to study the convergence speed in a statistical signal processing problem on filtering WSS vector processes.

The paper is organized as follows. In Section 2, we give several new mathematical results on block Toeplitz matrices. In Section 3, using the results obtained in Section 2, we give several new mathematical results on the correlation matrices of WSS, MA, and AR vector processes. In Section 4, we recall the low-complexity coding strategy presented in [4], and using the results obtained in Section 3, we study the asymptotic optimality of such a coding strategy when it is used to encode WSS, MA, and AR vector sources. In Section 4, we also study how the coding strategy considered performs when it is used to encode perturbed versions of those sources. Finally, in Section 5, some conclusions are presented.

## 2. Several New Results on Block Toeplitz Matrices

In this section, we present new results on the product of block Toeplitz matrices, on the inverse of a block Toeplitz matrix, and on block circulant matrices. These results will be used in Section 3. We begin by introducing some notation.

### 2.1. Notation

In this paper, N, Z, R, and C denote the set of natural numbers (that is, the set of positive integers), the set of integer numbers, the set of real numbers, and the set of complex numbers, respectively. $C^{M\times N}$ is the set of all M×N complex matrices. $I_{N}$ stands for the N×N identity matrix. $0_{M\times N}$ denotes the M×N zero matrix. $V_{n}$ is the n×n Fourier unitary matrix, i.e.,

$${\left[V_{n}\right]}_{j,k}=\frac{1}{\sqrt{n}}e^{-\frac{2\pi (j-1)(k-1)}{n}i},\;j,k\in \left\{1,\ldots ,n\right\},$$

with i being the imaginary unit. We denote by $\lambda_{1}\left(A\right),\ldots ,\lambda_{n}\left(A\right)$ the eigenvalues of an n×n Hermitian matrix *A* arranged in decreasing order. ∗ denotes the conjugate transpose. ⊗ is the Kronecker product. ${∥\cdot ∥}_{2}$ and ${∥\cdot ∥}_{F}$ are the spectral norm and the Frobenius norm, respectively.

If n∈N and $A_{j}\in C^{M\times N}$ for all j∈{1,…,n}, then $\mathrm{diag}(A_{1},\ldots ,A_{n})$ is the n×n block diagonal matrix whose M×N blocks are given by:

$${\left[\mathrm{diag}\left(A_{1},\ldots ,A_{n}\right)\right]}_{j,k}=\delta_{j,k}A_{j},\;j,k\in \left\{1,\ldots ,n\right\},$$

where δ is the Kronecker delta. We also denote by ${\mathrm{diag}}_{1\leq k\leq n}\left(A_{k}\right)$ the matrix $\mathrm{diag}(A_{1},\ldots ,A_{n})$.

If n∈N and $F:R\to C^{M\times N}$ is a continuous 2π-periodic function, $T_{n}\left(F\right)$ stands for the n×n block Toeplitz matrix generated by *F* whose M×N blocks are given by:

$${\left[T_{n}\left(F\right)\right]}_{j,k}=F_{j-k},\;j,k\in \left\{1,\ldots ,n\right\},$$

where ${\left\{F_{k}\right\}}_{k\in Z}$ is the sequence of Fourier coefficients of *F*, that is,

$$F_{k}=\frac{1}{2\pi }\int_{0}^{2\pi }e^{-k\omega i}F\left(\omega \right)d\omega \;\forall k\in Z.$$

We denote by $C_{n}\left(F\right)$ the n×n block circulant matrix with M×N blocks defined as:

$$C_{n}\left(F\right)=\left(V_{n}⊗I_{M}\right){\mathrm{diag}}_{1\leq k\leq n}\left(F\left(\frac{2\pi (k-1)}{n}\right)\right){\left(V_{n}⊗I_{N}\right)}^{*}.$$

If $A_{n}\in C^{nM\times nN}$, then $C_{A_{n}}$ is the n×n block circulant matrix with the M×N blocks given by:

$$C_{A_{n}}=\left(V_{n}⊗I_{M}\right){\mathrm{diag}}_{1\leq k\leq n}\left({\left[{\left(V_{n}⊗I_{M}\right)}^{*}A_{n}\left(V_{n}⊗I_{N}\right)\right]}_{k,k}\right){\left(V_{n}⊗I_{N}\right)}^{*}.$$

We denote by ${\hat{C}}_{n}\left(F\right)$ the n×n block circulant matrix with the M×N blocks defined as ${\hat{C}}_{n}\left(F\right)=C_{T_{n}\left(F\right)}$.

If F(ω) is Hermitian for all ω∈R (or equivalently, $T_{n}\left(F\right)$ is Hermitian for all n∈N (see, e.g., [6], Theorem 4.4), then infF denotes $\inf_{\omega \in [0,2\pi ]}\lambda_{N}\left(F\left(\omega \right)\right)$. We recall that (see [7], Proposition 3):

(1) $$\underset{n\in N}{\inf}\lambda_{nN}\left(T_{n}\left(F\right)\right)=\inf F=\min_{\omega \in [0,2\pi ]}\lambda_{N}\left(F\left(\omega \right)\right).$$

### 2.2. Product of Block Toeplitz Matrices

We begin this subsection with a result on the entries of the block Toeplitz matrices generated by the product of two functions, which is a direct consequence of the Parseval theorem.

**Lemma** **1.** *Consider two continuous 2π-periodic functions $F:R\to C^{M\times N}$ and $G:R\to C^{N\times K}$. Let ${\left\{F_{k}\right\}}_{k\in Z}$ and ${\left\{G_{k}\right\}}_{k\in Z}$ be the sequences of Fourier coefficients of F and G, respectively. Then:*

$${\left[T_{n}\left(FG\right)\right]}_{j,k}=\sum_{h=-\infty }^{\infty }F_{j-h}G_{h-k}$$

*for all n∈N and j,k∈{1,…,n}.*

**Proof.**  See Appendix A. □

We can now give a result on the product of two block Toeplitz matrices when one of them is generated by a trigonometric polynomial. We recall that an M×N trigonometric polynomial of degree p∈N∪{0} is a function $F:R\to C^{M\times N}$ of the form:

(2) $$F\left(\omega \right)=\sum_{k=-p}^{p}e^{k\omega i}A_{k}\;\forall \omega \in R,$$

where $A_{k}\in C^{M\times N}$ with |k|≤p. It can be easily proven (see, e.g., [6], Example 4.3) that the sequence of the Fourier coefficients ${\left\{F_{k}\right\}}_{k\in Z}$ of the continuous 2π-periodic function *F* in Equation (2) is given by:

$$F_{k}=\left\{\begin{array}{cc} A_{k}\; & \mathrm{if}\;|k|\leq p,\\ 0_{M\times N} & \mathrm{if}\;|k|>p. \end{array}\right.$$

**Lemma** **2.**  *Let F, G, ${\left\{F_{k}\right\}}_{k\in Z}$, and ${\left\{G_{k}\right\}}_{k\in Z}$ be as in Lemma 1.*

*1.*  *If F is a trigonometric polynomial of degree p, then:*
  (3) $${\left[T_{n}\left(F\right)T_{n}\left(G\right)-T_{n}\left(FG\right)\right]}_{j,k}=\left\{\begin{array}{cc} -\sum_{h=j-p}^{0}F_{j-h}G_{h-k} & if\;j\leq p,\\ 0_{M\times K} & if\;p+1\leq j\leq n-p,\\ -\sum_{h=n+1}^{j+p}F_{j-h}G_{h-k} & if\;j\geq n-p+1, \end{array}\right.$$
*and:*
  (4) $$∥T_{n}\left(F\right)T_{n}\left(G\right)-T_{n}{\left(FG\right)∥}_{F}\leq \sqrt{p\left(p+1\right)\left(\frac{1}{2\pi }\int_{0}^{2\pi }{∥F\left(\omega \right)∥}_{F}^{2}d\omega \right)\left(\frac{1}{2\pi }\int_{0}^{2\pi }{∥G\left(\omega \right)∥}_{F}^{2}d\omega \right)}$$
*for all n∈N and j,k∈{1,…,n}.*
*2.*  *If G is a trigonometric polynomial of degree q, then:*
  (5) $${\left[T_{n}\left(F\right)T_{n}\left(G\right)-T_{n}\left(FG\right)\right]}_{j,k}=\left\{\begin{array}{cc} -\sum_{h=k-q}^{0}F_{j-h}G_{h-k} & if\;k\leq q,\\ 0_{M\times K} & if\;q+1\leq k\leq n-q,\\ -\sum_{h=n+1}^{k+q}F_{j-h}G_{h-k} & if\;k\geq n-q+1, \end{array}\right.$$
*and:*
  (6) $$∥T_{n}\left(F\right)T_{n}\left(G\right)-T_{n}{\left(FG\right)∥}_{F}\leq \sqrt{q\left(q+1\right)\left(\frac{1}{2\pi }\int_{0}^{2\pi }{∥F\left(\omega \right)∥}_{F}^{2}d\omega \right)\left(\frac{1}{2\pi }\int_{0}^{2\pi }{∥G\left(\omega \right)∥}_{F}^{2}d\omega \right)}$$
*for all n∈N and j,k∈{1,…,n}.*
*3.*  *If F is a trigonometric polynomial of degree p and G is a trigonometric polynomial of degree q, then:*
  $$T_{n}\left(F\right)T_{n}\left(G\right)-T_{n}\left(FG\right)=\left(\begin{array}{ccc} \xi_{1}\left(F,G\right) & 0_{pM\times (n-2q)K} & 0_{pM\times qK}\\ 0_{(n-2p)M\times qK} & 0_{(n-2p)M\times (n-2q)K} & 0_{(n-2p)M\times qK}\\ 0_{pM\times qK} & 0_{pM\times (n-2q)K} & \xi_{2}\left(F,G\right) \end{array}\right)$$
*and:*
  (7) $$∥T_{n}\left(F\right)T_{n}\left(G\right)-T_{n}{\left(FG\right)∥}_{F}=\sqrt{∥\xi_{1}{\left(F,G\right)∥}_{F}^{2}+{∥\xi_{2}\left(F,G\right)∥}_{F}^{2}}$$
*for all n≥max{2p,2q}, where $\xi_{1}\left(F,G\right),\xi_{2}\left(F,G\right)\in C^{pM\times qK}$ are given by:*
  $${\left[\xi_{1}\left(F,G\right)\right]}_{j,k}=-\sum_{h=\max\{j-p,k-q\}}^{0}F_{j-h}G_{h-k}$$
*and:*
  $${\left[\xi_{2}\left(F,G\right)\right]}_{j,k}=-\sum_{h=1}^{\min\{j,k\}}F_{j-p-h}G_{h+q-k}$$
*for all j∈{1,…,p} and k∈{1,…,q}.*

**Proof.**  See Appendix B. □

### 2.3. Inverse of a Block Toeplitz Matrix

**Lemma** **3.**  *Let $F:R\to C^{N\times N}$ be a trigonometric polynomial of degree p.*

*1.*  *If F(ω) is invertible for all ω∈R and $\left\{T_{n}\left(F\right)\right\}$ is stable (i.e., $T_{n}\left(F\right)$ is invertible for all n∈N and $\left\{∥{\left(T_{n}\left(F\right)\right)}^{-1}{∥}_{2}\right\}$ is bounded), then:*
  $$∥\;{\left(T_{n}\left(F\right)\right)}^{-1}-T_{n}\left(F^{-1}\right){∥}_{F}\leq \underset{m\in N}{\sup}{∥{\left(T_{m}\left(F\right)\right)}^{-1}\;∥}_{2}\sqrt{p\left(p+1\right)\left(\frac{1}{2\pi }\int_{0}^{2\pi }{∥F\left(\omega \right)∥}_{F}^{2}d\omega \right)\left(\frac{1}{2\pi }\int_{0}^{2\pi }{∥{\left(\;F\left(\omega \right)\right)}^{-1}∥}_{F}^{2}d\omega \right)}$$
*for all n∈N.*
*2.*  *If F(ω) is positive definite for all ω∈R, then:*
  (8) $$∥{\left(T_{n}\left(F\right)\right)}^{-1}-T_{n}\left(F^{-1}\right){∥}_{F}\leq \frac{1}{\inf F}\sqrt{p\left(p+1\right)\left(\frac{1}{2\pi }\int_{0}^{2\pi }{∥F\left(\omega \right)∥}_{F}^{2}d\omega \right)\left(\frac{1}{2\pi }\int_{0}^{2\pi }{∥{\left(F\left(\omega \right)\right)}^{-1}∥}_{F}^{2}d\omega \right)}$$
*for all n∈N.*

**Proof.**  See Appendix C. □

### 2.4. Block Circulant Matrices

**Lemma** **4.** *Consider $A_{n},B_{n}\in C^{nM\times nN}$. Then:*

$$∥C_{A_{n}}-C_{B_{n}}{∥}_{F}\leq {∥A_{n}-B_{n}∥}_{F}$$

*and:*

(9) $$∥A_{n}-C_{A_{n}}{∥}_{F}\leq 2∥A_{n}-B_{n}{∥}_{F}+{∥B_{n}-C_{B_{n}}∥}_{F}.$$

*Moreover, if $B_{n}$ is an n×n block circulant matrix with M×N blocks, then:*

(10) $$C_{B_{n}}=B_{n}$$

*and:*

(11) $$∥A_{n}-C_{A_{n}}{∥}_{F}\leq 2{∥A_{n}-B_{n}∥}_{F}.$$

**Proof.**  See Appendix D. □

**Lemma** **5.** *Let $F:R\to C^{M\times N}$ be a trigonometric polynomial of degree p. Then:*

$$∥T_{n}\left(F\right)-{\hat{C}}_{n}{\left(F\right)∥}_{F}\leq {∥T_{n}\left(F\right)-C_{n}\left(F\right)∥}_{F}=\sqrt{\sum_{k=1}^{p}k\left(∥F_{k}{∥}_{F}^{2}+{∥F_{-k}∥}_{F}^{2}\right)}$$

*for all n>2p. Furthermore,*

$$\lim_{n\to \infty }∥T_{n}\left(F\right)-{\hat{C}}_{n}{\left(F\right)∥}_{F}=\lim_{n\to \infty }{∥T_{n}\left(F\right)-C_{n}\left(F\right)∥}_{F}.$$

**Proof.**  See Appendix E. □

## 3. Several New Results on the Correlation Matrices of Certain Random Vector Processes

Let $\left\{x_{n}\right\}$ be a (complex) random *N*-dimensional vector process, that is $x_{n}$ is a (complex) random *N*-dimensional (column) vector for all n∈N. In this section, we study the boundedness of the sequence $\left\{{∥E\left(x_{n:1}x_{n:1}^{*}\right)-C_{E\left(x_{n:1}x_{n:1}^{*}\right)}∥}_{F}\right\}$ when $\left\{x_{n}\right\}$ is a WSS, MA, or AR vector process, where:

$$x_{n:1}=\left(\begin{array}{c} x_{n}\\ x_{n-1}\\ \vdots \\ x_{1} \end{array}\right),\;n\in N,$$

and *E* denotes expectation.

### 3.1. WSS Vector Processes

In this subsection, we review the concept of the WSS vector process, and we prove that the sequence $\left\{{∥E\left(x_{n:1}x_{n:1}^{*}\right)-C_{E\left(x_{n:1}x_{n:1}^{*}\right)}∥}_{F}\right\}$ is bounded when $\left\{x_{n}\right\}$ is a WSS vector process whose power spectral density (PSD) is a trigonometric polynomial.

**Definition** **1.** Let $X:R\to C^{N\times N}$ be continuous and 2π-periodic. A random N-dimensional vector process $\left\{x_{n}\right\}$ is said to be WSS with PSD X if it has constant mean (i.e., $E\left(x_{n_{1}}\right)=E\left(x_{n_{2}}\right)$ for all $n_{1},n_{2}\in N)$ and $\left\{E\left(x_{n:1}x_{n:1}^{*}\right)\right\}=\left\{T_{n}\left(X\right)\right\}$.

**Lemma** **6.** If $\left\{x_{n}\right\}$ is a WSS vector process whose PSD is a trigonometric polynomial, then $\left\{{∥E\left(x_{n:1}x_{n:1}^{*}\right)-C_{E\left(x_{n:1}x_{n:1}^{*}\right)}∥}_{F}\right\}$ is bounded.

**Proof.**  This is a direct consequence of Lemma 5. □

### 3.2. VMA Processes

In this subsection, we review the concept of the MA vector (VMA) process, and we prove that the sequence $\left\{{∥E\left(x_{n:1}x_{n:1}^{*}\right)-C_{E\left(x_{n:1}x_{n:1}^{*}\right)}∥}_{F}\right\}$ is bounded when $\left\{x_{n}\right\}$ is a VMA process of finite order.

**Definition** **2.** *A zero-mean random N-dimensional vector process $\left\{x_{n}\right\}$ is said to be a VMA process if:*

(12) $$x_{n}=w_{n}+\sum_{k=1}^{n-1}G_{-k}w_{n-k}\;\forall n\in N,$$

*where $G_{-k}\in C^{N\times N}$ for all k∈N and $\left\{w_{n}\right\}$ is a zero-mean WSS N-dimensional vector process whose PSD is an N×N positive semidefinite matrix Λ. If there exists q∈N such that $G_{-k}=0_{N\times N}$ for all k>q, then $\left\{x_{n}\right\}$ is called a VMA process of (finite) order q or a VMA(q) process.*

**Lemma** **7.** If $\left\{x_{n}\right\}$ is a VMA(q) process as in Definition 2, then $\left\{{∥E\left(x_{n:1}x_{n:1}^{*}\right)-C_{E\left(x_{n:1}x_{n:1}^{*}\right)}∥}_{F}\right\}$ is bounded.

**Proof.**  See Appendix F. □

### 3.3. VAR Processes

In this subsection, we review the concept of the AR vector (VAR) process, and we study the boundedness of the sequence $\left\{{∥E\left(x_{n:1}x_{n:1}^{*}\right)-C_{E\left(x_{n:1}x_{n:1}^{*}\right)}∥}_{F}\right\}$ when $\left\{x_{n}\right\}$ is a VAR process of finite order.

**Definition** **3.** *A zero-mean random N-dimensional vector process $\left\{x_{n}\right\}$ is said to be a VAR process if:*

(13) $$x_{n}=w_{n}-\sum_{k=1}^{n-1}F_{-k}x_{n-k}\;\forall n\in N,$$

*where $F_{-k}\in C^{N\times N}$ for all k∈N and $\left\{w_{n}\right\}$ is a zero-mean WSS N-dimensional vector process whose PSD is an N×N positive definite matrix Λ. If there exists p∈N such that $F_{-k}=0_{N\times N}$ for all k>p, then $\left\{x_{n}\right\}$ is called a VAR process of (finite) order p or a VAR(p) process.*

**Lemma** **8.** Let $\left\{x_{n}\right\}$ be a VAR(p) process as in Definition 3. Suppose that $F\left(\omega \right)=I_{N}+\sum_{k=1}^{p}e^{-k\omega i}F_{-k}$ is invertible for all ω∈R and $\left\{∥{\left(T_{n}\left(F\right)\right)}^{-1}{∥}_{2}\right\}$ is bounded. Then, $\left\{{∥E\left(x_{n:1}x_{n:1}^{*}\right)-C_{E\left(x_{n:1}x_{n:1}^{*}\right)}∥}_{F}\right\}$ is bounded.

**Proof.**  See Appendix G. □

## 4. On the Asymptotic Optimality of a Low-Complexity Coding Strategy for Gaussian Vector Sources

### 4.1. Low-Complexity Coding Strategy Considered

In [1], Kolmogorov gave a formula for the RDF of a real zero-mean Gaussian *N*-dimensional vector x with a positive definite correlation matrix $E\left(xx^{⊤}\right)$, namely,

$$R_{x}\left(D\right)=\frac{1}{N}\sum_{k=1}^{N}\max\left\{0,\frac{1}{2}\ln\frac{\lambda_{k}\left(E\left(xx^{⊤}\right)\right)}{\theta }\right\}\;\forall D\in \left(0,\frac{\mathrm{tr}\left(E\left(xx^{⊤}\right)\right)}{N}\right],$$

where *⊤* stands for the transpose, tr denotes the trace, and θ is a real number satisfying:

$$D=\frac{1}{N}\sum_{k=1}^{N}\min\left\{\theta ,\lambda_{k}\left(E\left(xx^{⊤}\right)\right)\right\}.$$

We recall that $R_{x}\left(D\right)$ can be thought of as the minimum rate (measured in nats) at which x can be encoded (compressed) in order to be able to recover it with a mean squared error (MSE) per dimension no larger than a given distortion *D*, that is:

$$\frac{E\left({∥x-\tilde{x}∥}_{2}^{2}\right)}{N}\leq D,$$

where $\tilde{x}$ denotes the estimation of x.

If $D\in \left(0,\lambda_{N}\left(E\left(xx^{⊤}\right)\right)\right]$, an optimal coding strategy to achieve $R_{x}\left(D\right)$ is to encode ${\left[z\right]}_{1,1},\ldots ,{\left[z\right]}_{N,1}$ separately with $E(∥{\left[z\right]}_{k,1}-\tilde{{\left[z\right]}_{k,1}}{∥}_{2}^{2})\leq D$ for all k∈{1,…,N}, where $z=U^{⊤}x$ with *U* being a real orthogonal eigenvector matrix of $E\left(xx^{⊤}\right)$ (see [8], Corollary 1). Observe that in order to obtain *U*, we need to know the correlation matrix $E\left(xx^{⊤}\right)$. This coding strategy also requires an optimal coding method for real Gaussian random variables.

In [4], Theorem 3, we gave a low-complexity coding strategy for any Gaussian *N*-dimensional vector source $\left\{x_{n}\right\}$. According to that strategy, to encode a finite-length data block $x_{n:1}$ of such a source, we first compute the block discrete Fourier transform (DFT) of $x_{n:1}$:

(14) $$y_{n:1}=\left(V_{n}^{*}⊗I_{N}\right)x_{n:1},$$

and then, we encode $y_{\left\lceil \frac{n}{2}\right\rceil },\ldots ,y_{n}$ separately (i.e., if *n* is even, we encode $y_{\frac{n}{2}},\hat{y_{\frac{n}{2}+1}},\ldots ,\hat{y_{n-1}},y_{n}$ separately, and if *n* is odd, we encode $\hat{y_{\frac{n+1}{2}}},\ldots ,\hat{y_{n-1}},y_{n}$ separately) with:

$$\frac{E\left({∥\hat{y_{k}}-\tilde{\hat{y_{k}}}∥}_{2}^{2}\right)}{2N}\leq \frac{D}{2},\;k\in \left\{\left\lceil \frac{n}{2}\right\rceil ,\ldots ,n-1\right\}\backslash \left\{\frac{n}{2}\right\},$$

and:

$$\frac{E\left({∥y_{k}-\tilde{y_{k}}∥}_{2}^{2}\right)}{N}\leq D,\;k\in \left\{\frac{n}{2},n\right\}\cap N,$$

where ⌈x⌉ denotes the smallest integer higher than or equal to x∈R and:

z^=Re(z)Im(z)=Re([z]1,1)⋮Re([z]N,1)Im([z]1,1)⋮Im([z]N,1)∀z∈CN×1

with Re and Im being the real part and the imaginary part of a complex number, respectively.

As our coding strategy requires the computation of the block DFT, its computational complexity is O(nNlogn) whenever the fast Fourier transform (FFT) algorithm is used. We recall that the computational complexity of the optimal coding strategy for $x_{n:1}$ is $O(n^{2}N^{2})$ since it requires the computation of $U_{n}^{⊤}x_{n:1}$, where $U_{n}$ is a real orthogonal eigenvector matrix of $E\left(x_{n:1}x_{n:1}^{⊤}\right)$. Observe that such an eigenvector matrix $U_{n}$ also needs to be computed, which further increases the complexity. Hence, the main advantage of our coding strategy is that it notably reduces the computational complexity of coding $x_{n:1}$. Moreover, our coding strategy does not require the knowledge of $E\left(x_{n:1}x_{n:1}^{⊤}\right)$. It only requires the knowledge of $E\left(\hat{y_{k}}{\hat{y_{k}}}^{⊤}\right)$, with $k\in \{\left\lceil \frac{n}{2}\right\rceil ,\ldots ,n\}$.

We finish this subsection by reviewing a result that provides an upper bound for the distance between $R_{x_{n:1}}\left(D\right)$ and the rate of our coding strategy ${\tilde{R}}_{x_{n:1}}\left(D\right)$ (see [4], Theorem 3).

**Theorem** **1.** *Consider n,N∈N. Let $x_{k}$ be a random N-dimensional vector for all k∈{1,…,n}. Suppose that $x_{n:1}$ is a real zero-mean Gaussian vector with a positive definite correlation matrix (or equivalently, $\lambda_{nN}\left(E\left(x_{n:1}x_{n:1}^{⊤}\right)\right)>0$). Let $y_{n:1}$ be the random vector given by Equation (14). If $D\in \left(0,\lambda_{nN}\left(E\left(x_{n:1}x_{n:1}^{⊤}\right)\right)\right]$, then:*

(15) $$\begin{array}{c} 0\leq {\tilde{R}}_{x_{n:1}}\left(D\right)-R_{x_{n:1}}\left(D\right)\leq \frac{1}{2}\ln\left(1+\frac{{∥E\left(x_{n:1}x_{n:1}^{⊤}\right)-C_{E\left(x_{n:1}x_{n:1}^{⊤}\right)}∥}_{F}}{\sqrt{nN}\lambda_{nN}\left(E\left(x_{n:1}x_{n:1}^{⊤}\right)\right)}\right), \end{array}$$

*where:*

$${\tilde{R}}_{x_{n:1}}\left(D\right)=\left\{\begin{array}{cc} \frac{R_{y_{\frac{n}{2}}}\left(D\right)+2\sum_{k=\frac{n}{2}+1}^{n-1}R_{\hat{y_{k}}}\left(\frac{D}{2}\right)+R_{y_{n}}\left(D\right)}{n} & \mathrm{if}\;n\;\mathrm{is}\;\mathrm{even},\;\\ \frac{2\sum_{k=\frac{n+1}{2}}^{n-1}R_{\hat{y_{k}}}\left(\frac{D}{2}\right)+R_{y_{n}}\left(D\right)}{n} & \mathrm{if}\;n\;\mathrm{is}\;\mathrm{odd}. \end{array}\right.$$

### 4.2. On the Asymptotic Optimality of the Low-Complexity Coding Strategy Considered

In this subsection, we study the asymptotic optimality of our coding strategy for Gaussian vector sources. We begin by presenting a new result that provides a sufficient condition for the source to make such a coding strategy asymptotically optimal.

**Theorem** **2.** *Let $\left\{x_{n}\right\}$ be a real zero-mean Gaussian N-dimensional vector process. Suppose that $\inf_{n\in N}\lambda_{nN}\left(E\left(x_{n:1}x_{n:1}^{⊤}\right)\right)>0$ and $\lim_{n\to \infty }\frac{{∥E\;\left(x_{n:1}x_{n:1}^{⊤}\right)-C_{E\left(x_{n:1}x_{n:1}^{⊤}\right)}∥}_{F}}{\sqrt{n}}=0$. If $D\in \left(0,\inf_{n\in N}\lambda_{nN}\left(E\left(x_{n:1}x_{n:1}^{⊤}\right)\right)\right]$, then:*

$$\lim_{n\to \infty }\left({\tilde{R}}_{x_{n:1}}\left(D\right)-R_{x_{n:1}}\left(D\right)\right)=0.$$

*Hence, if $\left\{R_{x_{n:1}}\left(D\right)\right\}$ is convergent, then:*

(16) $$\lim_{n\to \infty }{\tilde{R}}_{x_{n:1}}\left(D\right)=\lim_{n\to \infty }R_{x_{n:1}}\left(D\right).$$

**Proof.**  From Equation (15), we have:

$$\begin{array}{cc} 0\; & \leq \;{\tilde{R}}_{x_{n:1}}\left(D\right)-R_{x_{n:1}}\left(D\right)\leq \frac{1}{2}\ln\left(1+\frac{{∥E\left(x_{n:1}x_{n:1}^{⊤}\right)-C_{E\left(x_{n:1}x_{n:1}^{⊤}\right)}∥}_{F}}{\sqrt{nN}\inf_{m\in N}\lambda_{mN}\left(E\left(x_{m:1}x_{m:1}^{⊤}\right)\right)}\right)\;\forall n\in N, \end{array}$$

and therefore, Theorem 2 is proven. □

We recall that $\lim_{n\to \infty }R_{x_{n:1}}\left(D\right)$ is the RDF of the source $\left\{x_{n}\right\}$.

In [4], Theorem 4, we gave a more restrictive sufficient condition for the source to make the coding strategy considered asymptotically optimal. Specifically, in [4], Theorem 4, we proved that Equation (16) holds if $\left\{x_{n}\right\}$ is AWSS. However, the convergence speed of the rate of the coding strategy considered (i.e., how fast the rate of the coding strategy tends to the RDF of the AWSS vector source) was not studied in [4]. We now study the convergence speed of the rate of such a coding strategy when it is used to encode the most relevant vector sources, namely WSS vector sources, VMA sources, and VAR sources. It should be mentioned that this convergence speed depends on the sequence $\left\{{∥E\left(x_{n:1}x_{n:1}^{⊤}\right)-C_{E\left(x_{n:1}x_{n:1}^{⊤}\right)}∥}_{F}\right\}$ whose boundedness is studied in Section 3 for these three types of vector sources.

**Theorem** **3.** *Let $\left\{x_{n}\right\}$ be a real zero-mean Gaussian WSS N-dimensional vector process whose PSD X is a trigonometric polynomial. Suppose that infX>0 (or equivalently, det(X(ω))≠0 for all ω∈R). If $D\in \left(0,\inf X\right]$, there exists K∈[0,∞) such that:*

(17) $$\begin{array}{cc} 0 & \leq {\tilde{R}}_{x_{n:1}}\left(D\right)-R_{x_{n:1}}\left(D\right)\leq \frac{1}{2}\ln\left(1+\frac{K}{\sqrt{n}}\right)\;\forall n\in N. \end{array}$$

**Proof.**  As $\left\{T_{n}\left(X\right)\right\}=\left\{E\left(x_{n:1}x_{n:1}^{*}\right)\right\}$, $T_{n}\left(X\right)$ is positive semidefinite for all n∈N. Consequently, from [7], Proposition 3, X(ω) is positive semidefinite for all ω∈R. Therefore, applying Equation (1), infX>0 if and only if det(X(ω))≠0 for all ω∈R. Equation (17) is a direct consequence of Equation (1), Theorem 1, and Lemma 6. □

**Theorem** **4.** *Let $\left\{x_{n}\right\}$ be a VMA(q) process as in Definition 2. Suppose that det(Λ)≠0 and $\left\{∥{\left(T_{n}\left(G\right)\right)}^{-1}{∥}_{2}\right\}$ is bounded with $G\left(\omega \right)=I_{N}+\sum_{k=1}^{q}e^{-k\omega i}G_{-k}$ for all ω∈R. If $\left\{x_{n}\right\}$ is real and Gaussian, and $D\in \left(0,\inf_{n\in N}\lambda_{nN}\left(E\left(x_{n:1}x_{n:1}^{⊤}\right)\right)\right]$, there exists K∈[0,∞) such that:*

$$\begin{array}{cc} 0 & \leq {\tilde{R}}_{x_{n:1}}\left(D\right)-R_{x_{n:1}}\left(D\right)\leq \frac{1}{2}\ln\left(1+\frac{K}{\sqrt{n}}\right)\;\forall n\in N. \end{array}$$

**Proof.**  Since $\det(T_{n}\left(G\right))=1$ for all n∈N, from Equation (A3), we have:

$$\begin{array}{cc} \; & \lambda_{nN}\left(E\left(x_{n:1}x_{n:1}^{⊤}\right)\right)=\frac{1}{{∥{\left(E\left(x_{n:1}x_{n:1}^{⊤}\right)\right)}^{-1}∥}_{2}}=\frac{1}{{∥{\left(T_{n}\left(G\right)T_{n}\left(\Lambda \right){\left(T_{n}\left(G\right)\right)}^{*}\right)}^{-1}∥}_{2}}\\ \; & \;\;\;\;\;\;\;\;\;\;\;=\frac{1}{{∥{\left({\left(T_{n}\left(G\right)\right)}^{-1}\right)}^{*}T_{n}\left(\Lambda^{-1}\right){\left(T_{n}\left(G\right)\right)}^{-1}∥}_{2}}\geq \frac{1}{{∥{\left({\left(T_{n}\left(G\right)\right)}^{-1}\right)}^{*}∥}_{2}{∥T_{n}\left(\Lambda^{-1}\right)∥}_{2}{∥{\left(T_{n}\left(G\right)\right)}^{-1}∥}_{2}}\\ \; & \;\;\;\;\;\;\;\;\;\;\;=\frac{\lambda_{N}\left(\Lambda \right)}{{∥{\left(T_{n}\left(G\right)\right)}^{-1}∥}_{2}^{2}}\geq \frac{\lambda_{N}\left(\Lambda \right)}{{\left(\sup_{m\in N}{∥{\left(T_{m}\left(G\right)\right)}^{-1}∥}_{2}\right)}^{2}}>0\;\forall n\in N. \end{array}$$

Hence, $\inf_{n\in N}\lambda_{nN}\left(E\left(x_{n:1}x_{n:1}^{⊤}\right)\right)>0$. Theorem 1 and Lemma 7 prove Theorem 4. □

**Theorem** **5.** *Let $\left\{x_{n}\right\}$ be a VAR(p) process as in Definition 3. Suppose that $F\left(\omega \right)=I_{N}+\sum_{k=1}^{p}e^{-k\omega i}F_{-k}$ is invertible for all ω∈R and $\left\{∥{\left(T_{n}\left(F\right)\right)}^{-1}{∥}_{2}\right\}$ is bounded. If $\left\{x_{n}\right\}$ is real and Gaussian and $D\in \left(0,\inf_{n\in N}\lambda_{nN}\left(E\left(x_{n:1}x_{n:1}^{⊤}\right)\right)\right]$, there exists K∈[0,∞) such that:*

$$\begin{array}{cc} 0 & \leq {\tilde{R}}_{x_{n:1}}\left(D\right)-R_{x_{n:1}}\left(D\right)\leq \frac{1}{2}\ln\left(1+\frac{K}{\sqrt{n}}\right)\;\forall n\in N. \end{array}$$

**Proof.**  As $\det(T_{n}\left(F\right))=1$ for all n∈N, applying Equation (A4) and [6], Theorem 4.3, yields:

$$\begin{array}{cc} \lambda_{nN}\left(E\left(x_{n:1}x_{n:1}^{⊤}\right)\right) & =\frac{1}{{∥{\left(E\left(x_{n:1}x_{n:1}^{⊤}\right)\right)}^{-1}∥}_{2}}=\frac{1}{{∥{\left({\left(T_{n}\left(F\right)\right)}^{-1}T_{n}\left(\Lambda \right){\left({\left(T_{n}\left(F\right)\right)}^{*}\right)}^{-1}\right)}^{-1}∥}_{2}}\\ \; & =\frac{1}{{∥{\left(T_{n}\left(F\right)\right)}^{*}T_{n}\left(\Lambda^{-1}\right)T_{n}\left(F\right)∥}_{2}}\geq \frac{1}{{∥{\left(T_{n}\left(F\right)\right)}^{*}∥}_{2}{∥T_{n}\left(\Lambda^{-1}\right)∥}_{2}{∥T_{n}\left(F\right)∥}_{2}}\\ \; & =\frac{\lambda_{N}\left(\Lambda \right)}{{∥T_{n}\left(F\right)∥}_{2}^{2}}\geq \frac{\lambda_{N}\left(\Lambda \right)}{{\left(\sup_{m\in N}{∥T_{m}\left(F\right)∥}_{2}\right)}^{2}}>0\;\forall n\in N. \end{array}$$

Thus, $\inf_{n\in N}\lambda_{nN}\left(E\left(x_{n:1}x_{n:1}^{⊤}\right)\right)>0$. Theorem 1 and Lemma 8 prove Theorem 5. □

### 4.3. On How the Low-Complexity Coding Strategy Considered Performs under Perturbations

In this subsection, we study how the low-complexity coding strategy considered performs when it is used to encode a perturbed version, $\left\{z_{n}\right\}$, of a WSS, MA, or AR vector source $\left\{x_{n}\right\}$. Observe that if $\left\{{∥E\left(z_{n:1}z_{n:1}^{⊤}\right)-E\left(x_{n:1}x_{n:1}^{⊤}\right)∥}_{F}\right\}$ is bounded, from Equation (9), we conclude that our coding strategy can also be used to optimally encode $\left\{z_{n}\right\}$, and the convergence speed of the rate remains unaltered.

We now present three numerical examples that show how the coding strategy considered performs in the presence of a perturbation. In all of them, N=2 and:

$$E\left(z_{n:1}z_{n:1}^{⊤}\right)=E\left(x_{n:1}x_{n:1}^{⊤}\right)+\left(\begin{array}{cc} 0_{2n-2\times 2n-2} & 0_{2n-2\times 2}\\ 0_{2\times 2n-2} & I_{2} \end{array}\right)\;\forall n\in N.$$

Obviously, $\left\{{∥E\left(z_{n:1}z_{n:1}^{⊤}\right)-E\left(x_{n:1}x_{n:1}^{⊤}\right)∥}_{F}\right\}$ is bounded since ${∥E\left(z_{n:1}z_{n:1}^{⊤}\right)-E\left(x_{n:1}x_{n:1}^{⊤}\right)∥}_{F}=\sqrt{2}$ for all n∈N. The three vector sources $\left\{x_{n}\right\}$ considered in our numerical examples are the zero-mean WSS vector source in [9], Section 4, the VMA(1) source in [10], Example 2.1, and the VAR(1) source in [10], Example 2.3. In [9], Section 4, the Fourier coefficients of the PSD *X* are:

$$X_{0}=\left(\begin{array}{cc} 2.0002 & 0.7058\\ 0.7058 & 2.0000 \end{array}\right),\;X_{-1}=X_{1}^{*}=\left(\begin{array}{cc} -0.3542 & 0.1016\\ 0.1839 & -0.2524 \end{array}\right),\;X_{-2}=X_{2}^{*}=\left(\begin{array}{cc} -0.0923 & 0.0153\\ 0.1490 & 0.0696 \end{array}\right),$$

$$X_{-3}=X_{3}^{*}=\left(\begin{array}{cc} -0.1443 & -0.0904\\ 0.0602 & 0.0704 \end{array}\right),\;X_{-4}=X_{4}^{*}=\left(\begin{array}{cc} -0.0516 & -0.0603\\ 0 & 0 \end{array}\right),$$

and $X_{k}=0_{2\times 2}$ with |k|>4. In [10], Example 2.1, $G_{-1}$ and Λ are given by:

(18) $$\left(\begin{array}{cc} -0.8 & -0.7\\ 0.4 & -0.6 \end{array}\right)$$

and:

(19) $$\left(\begin{array}{cc} 4 & 1\\ 1 & 2 \end{array}\right),$$

respectively. In [10], Example 2.3, $F_{-1}$ and Λ are given by Equations (18) and (19), respectively.

**Figure 1:**
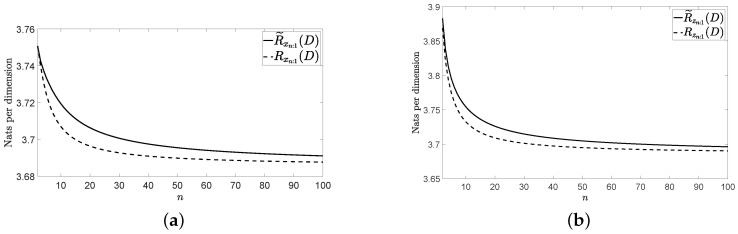
Rates for the considered wide sense stationary (WSS) vector source: (**a**) without perturbation and (**b**) with perturbation.

**Figure 2:**
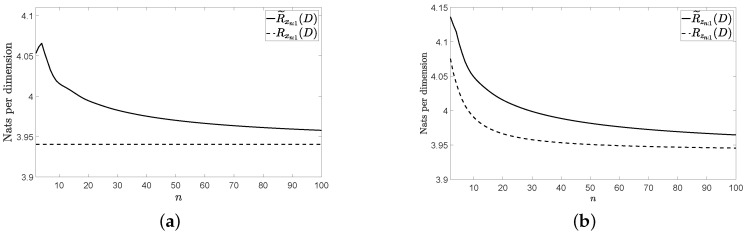
Rates for the considered VMA(1) source: (**a**) without perturbation and (**b**) with perturbation.

**Figure 3:**
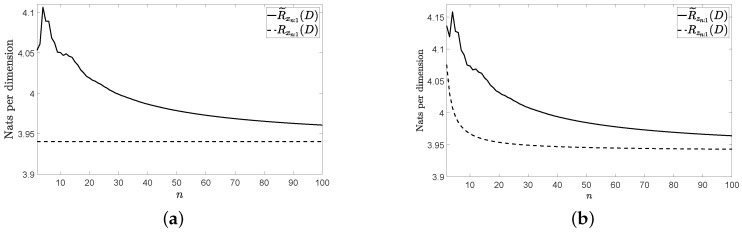
Rates for the considered VAR(1) source: (**a**) without perturbation and (**b**) with perturbation.

Figure 1a, Figure 2a and Figure 3a show $R_{x_{n:1}}\left(D\right)$ and ${\tilde{R}}_{x_{n:1}}\left(D\right)$ for the three vector sources $\left\{x_{n}\right\}$ considered by assuming that they are Gaussian. Figure 1b, Figure 2b and Figure 3b show $R_{z_{n:1}}\left(D\right)$ and ${\tilde{R}}_{z_{n:1}}\left(D\right)$ for these three vector sources. In Figure 1, Figure 2 and Figure 3, n≤100 and D=0.001. The figures bear the evidence of the fact that the rate of the low-complexity coding strategy considered tends to the RDF of the source even in the presence of a perturbation.

## 5. Conclusions

In [4], we proposed a low-complexity coding strategy to encode finite-length data blocks of any Gaussian vector source. In this paper, we proved that the convergence speed of the rate of our coding strategy is $O\left(\frac{1}{\sqrt{n}}\right)$ when it is used to encode the most relevant vector sources, namely WSS, MA, and AR vector sources. This means that the rate of our coding strategy will be close enough to the RDF of the source even if the length *n* of the data blocks is relatively small. Therefore, we conclude that our coding strategy is not only low-complexity and asymptotically optimal, but also low-latency. These three features make our coding strategy very useful in practical coding applications. 

## Appendix A. Proof of Lemma 1

**Proof.** Fix n∈N and j,k∈{1,…,n}. As $F:R\to C^{M\times N}$ and $G:R\to C^{N\times K}$ are continuous and 2π-periodic, $FG:R\to C^{M\times K}$ is also continuous and 2π-periodic. Applying the Parseval theorem (see, e.g., [11], p. 191) yields:

$$\begin{array}{cc} {\left[{\left[T_{n}\left(FG\right)\right]}_{j,k}\right]}_{r,s} & ={\left[\frac{1}{2\pi }\int_{0}^{2\pi }e^{-(j-k)\omega i}F\left(\omega \right)G\left(\omega \right)d\omega \right]}_{r,s}\\ \; & =\frac{1}{2\pi }\int_{0}^{2\pi }e^{-(j-k)\omega i}{\left[F\left(\omega \right)G\left(\omega \right)\right]}_{r,s}d\omega \\ \; & =\frac{1}{2\pi }\int_{0}^{2\pi }e^{(k-j)\omega i}\sum_{t=1}^{N}{\left[F\left(\omega \right)\right]}_{r,t}{\left[G\left(\omega \right)\right]}_{t,s}d\omega \\ \; & =\sum_{t=1}^{N}\frac{1}{2\pi }\int_{0}^{2\pi }e^{(k-j)\omega i}{\left[F\left(\omega \right)\right]}_{r,t}{\left[G\left(\omega \right)\right]}_{t,s}d\omega \\ \; & =\sum_{t=1}^{N}\frac{1}{2\pi }\int_{0}^{2\pi }{\left[G\left(\omega \right)\right]}_{t,s}e^{k\omega i}{\left[F\left(\omega \right)\right]}_{r,t}e^{-j\omega i}d\omega \\ \; & =\sum_{t=1}^{N}\sum_{h=-\infty }^{\infty }\left(\left(\frac{1}{2\pi }\int_{0}^{2\pi }e^{-h\omega i}{\left[G\left(\omega \right)\right]}_{t,s}e^{k\omega i}d\omega \right)\bar{\left(\frac{1}{2\pi }\int_{0}^{2\pi }e^{-h\omega i}\bar{{\left[F\left(\omega \right)\right]}_{r,t}e^{-j\omega i}}d\omega \right)}\right)\\ \; & =\sum_{t=1}^{N}\sum_{h=-\infty }^{\infty }\left(\left(\frac{1}{2\pi }\int_{0}^{2\pi }e^{-h\omega i}{\left[G\left(\omega \right)\right]}_{t,s}e^{k\omega i}d\omega \right)\left(\frac{1}{2\pi }\int_{0}^{2\pi }e^{h\omega i}{\left[F\left(\omega \right)\right]}_{r,t}e^{-j\omega i}d\omega \right)\right)\\ \; & =\sum_{t=1}^{N}\sum_{h=-\infty }^{\infty }\left(\left(\frac{1}{2\pi }\int_{0}^{2\pi }e^{-(j-h)\omega i}{\left[F\left(\omega \right)\right]}_{r,t}d\omega \right)\left(\frac{1}{2\pi }\int_{0}^{2\pi }e^{-(h-k)\omega i}{\left[G\left(\omega \right)\right]}_{t,s}d\omega \right)\right)\\ \; & =\sum_{t=1}^{N}\sum_{h=-\infty }^{\infty }\left({\left[\frac{1}{2\pi }\int_{0}^{2\pi }e^{-(j-h)\omega i}F\left(\omega \right)d\omega \right]}_{r,t}{\left[\frac{1}{2\pi }\int_{0}^{2\pi }e^{-(h-k)\omega i}G\left(\omega \right)d\omega \right]}_{t,s}\right)\\ \; & =\sum_{t=1}^{N}\sum_{h=-\infty }^{\infty }\left({\left[F_{j-h}\right]}_{r,t}{\left[G_{h-k}\right]}_{t,s}\right)\\ \; & =\sum_{h=-\infty }^{\infty }\sum_{t=1}^{N}\left({\left[F_{j-h}\right]}_{r,t}{\left[G_{h-k}\right]}_{t,s}\right)\\ \; & =\sum_{h=-\infty }^{\infty }{\left[F_{j-h}G_{h-k}\right]}_{r,s}\\ \; & ={\left[\sum_{h=-\infty }^{\infty }F_{j-h}G_{h-k}\right]}_{r,s} \end{array}$$

for all r∈{1,…,M} and s∈{1,…,K}. □

## Appendix B. Proof of Lemma 2

**Proof.** (1) Fix n∈N. As $F_{r}=0_{M\times N}$ with |r|>p, from Lemma 1, we have:

$$\begin{array}{cc} {\left[T_{n}\left(F\right)T_{n}\left(G\right)-T_{n}\left(FG\right)\right]}_{j,k} & ={\left[T_{n}\left(F\right)T_{n}\left(G\right)\right]}_{j,k}-{\left[T_{n}\left(FG\right)\right]}_{j,k}\\ \; & =\sum_{h=1}^{n}{\left[T_{n}\left(F\right)\right]}_{j,h}{\left[T_{n}\left(G\right)\right]}_{h,k}-\sum_{h=-\infty }^{\infty }F_{j-h}G_{h-k}\\ \; & =\sum_{h=1}^{n}F_{j-h}G_{h-k}-\lim_{H\to \infty }\left(\sum_{h=-H}^{H}F_{j-h}G_{h-k}\right)\\ \; & =-\lim_{H\to \infty }\left(\sum_{h=-H}^{0}F_{j-h}G_{h-k}+\sum_{h=n+1}^{H}F_{j-h}G_{h-k}\right)\\ \; & =-\sum_{h=j-p}^{0}F_{j-h}G_{h-k}-\sum_{h=n+1}^{j+p}F_{j-h}G_{h-k}, \end{array}$$

and consequently, Equation (3) holds for all j,k∈{1,…,n}. Applying Equation (3), the Schwarz inequality (see, e.g., [11], p. 15), the Parseval theorem for continuous matrix-valued functions (see, e.g., [6], p. 208), and the well-known formula for the partial sums of the arithmetic series yields:

$$\begin{array}{c} ∥T_{n}\left(F\right)T_{n}\left(G\right)-T_{n}{\left(FG\right)∥}_{F}^{2}\\ =\sum_{j=1}^{n}\sum_{k=1}^{n}{∥{\left[T_{n}\left(F\right)T_{n}\left(G\right)-T_{n}\left(FG\right)\right]}_{j,k}∥}_{F}^{2}\\ =\sum_{j=1}^{p}\sum_{k=1}^{n}{∥-\sum_{h=j-p}^{0}F_{j-h}G_{h-k}∥}_{F}^{2}+\sum_{j=n-p+1}^{n}\sum_{k=1}^{n}{∥-\sum_{h=n+1}^{j+p}F_{j-h}G_{h-k}∥}_{F}^{2}\\ =\sum_{j=1}^{p}\sum_{k=1}^{n}{∥\sum_{h=j-p}^{0}F_{j-h}G_{h-k}∥}_{F}^{2}+\sum_{j=n-p+1}^{n}\sum_{k=1}^{n}{∥\sum_{h=n+1}^{j+p}F_{j-h}G_{h-k}∥}_{F}^{2}\\ \leq \sum_{j=1}^{p}\sum_{k=1}^{n}{\left(\sum_{h=j-p}^{0}{∥F_{j-h}G_{h-k}∥}_{F}\right)}^{2}+\sum_{j=n-p+1}^{n}\sum_{k=1}^{n}{\left(\sum_{h=n+1}^{j+p}{∥F_{j-h}G_{h-k}∥}_{F}\right)}^{2}\\ \leq \sum_{j=1}^{p}\sum_{k=1}^{n}{\left(\sum_{h=j-p}^{0}∥F_{j-h}{∥}_{F}{∥G_{h-k}∥}_{F}\right)}^{2}+\sum_{j=n-p+1}^{n}\sum_{k=1}^{n}{\left(\sum_{h=n+1}^{j+p}∥F_{j-h}{∥}_{F}{∥G_{h-k}∥}_{F}\right)}^{2}\\ \leq \sum_{j=1}^{p}\sum_{k=1}^{n}\sum_{h=j-p}^{0}∥F_{j-h}{∥}_{F}^{2}\sum_{l=j-p}^{0}∥G_{l-k}{∥}_{F}^{2}+\sum_{j=n-p+1}^{n}\sum_{k=1}^{n}\sum_{h=n+1}^{j+p}∥F_{j-h}{∥}_{F}^{2}\sum_{l=n+1}^{j+p}{∥G_{l-k}∥}_{F}^{2}\\ =\sum_{j=1}^{p}\sum_{h=j-p}^{0}∥F_{j-h}{∥}_{F}^{2}\sum_{l=j-p}^{0}\sum_{k=1}^{n}∥G_{l-k}{∥}_{F}^{2}+\sum_{j=n-p+1}^{n}\sum_{h=n+1}^{j+p}∥F_{j-h}{∥}_{F}^{2}\sum_{l=n+1}^{j+p}\sum_{k=1}^{n}{∥G_{l-k}∥}_{F}^{2}\\ \leq \sum_{j=1}^{p}\sum_{h=j-p}^{0}∥F_{j-h}{∥}_{F}^{2}\sum_{l=j-p}^{0}\frac{1}{2\pi }\int_{0}^{2\pi }{∥G\left(\omega \right)∥}_{F}^{2}d\omega +\sum_{j=n-p+1}^{n}\sum_{h=n+1}^{j+p}∥F_{j-h}{∥}_{F}^{2}\sum_{l=n+1}^{j+p}\frac{1}{2\pi }\int_{0}^{2\pi }{∥G\left(\omega \right)∥}_{F}^{2}d\omega \\ =\frac{1}{2\pi }\int_{0}^{2\pi }{∥G\left(\omega \right)∥}_{F}^{2}d\omega \left(\sum_{j=1}^{p}\left(p-j+1\right)\sum_{h=j-p}^{0}∥F_{j-h}{∥}_{F}^{2}+\sum_{j=n-p+1}^{n}\left(j+p-n\right)\sum_{h=n+1}^{j+p}{∥F_{j-h}∥}_{F}^{2}\right)\\ \leq \frac{1}{2\pi }\int_{0}^{2\pi }{∥G\left(\omega \right)∥}_{F}^{2}d\omega \left(\sum_{j=1}^{p}\left(p-j+1\right)\frac{1}{2\pi }\int_{0}^{2\pi }{∥F\left(\omega \right)∥}_{F}^{2}d\omega +\;\;\;\sum_{j=n-p+1}^{n}\left(j+p-n\right)\frac{1}{2\pi }\int_{0}^{2\pi }{∥F\left(\omega \right)∥}_{F}^{2}d\omega \right)\\ =\left(\frac{1}{2\pi }\int_{0}^{2\pi }{∥F\left(\omega \right)∥}_{F}^{2}d\omega \right)\left(\frac{1}{2\pi }\int_{0}^{2\pi }{∥G\left(\omega \right)∥}_{F}^{2}d\omega \right)\left(\sum_{j=1}^{p}\left(p-j+1\right)+\sum_{j=n-p+1}^{n}\left(j+p-n\right)\right)\\ =\left(\frac{1}{2\pi }\int_{0}^{2\pi }{∥F\left(\omega \right)∥}_{F}^{2}d\omega \right)\left(\frac{1}{2\pi }\int_{0}^{2\pi }{∥G\left(\omega \right)∥}_{F}^{2}d\omega \right)\left(\frac{p(p+1)}{2}+\frac{p(1+p)}{2}\right), \end{array}$$

and therefore, Equation (4) is proven. (2) Fix n∈N. As $G_{r}=0_{N\times K}$ with |r|>q, from Lemma 1, we obtain:

$$\begin{array}{cc} {\left[T_{n}\left(F\right)T_{n}\left(G\right)-T_{n}\left(FG\right)\right]}_{j,k} & =-\lim_{H\to \infty }\left(\sum_{h=-H}^{0}F_{j-h}G_{h-k}+\sum_{h=n+1}^{H}F_{j-h}G_{h-k}\right)\\ \; & =-\sum_{h=k-q}^{0}F_{j-h}G_{h-k}-\;\;\sum_{h=n+1}^{k+q}F_{j-h}G_{h-k}, \end{array}$$

and hence, Equation (5) holds for all j,k∈{1,…,n}. Since *G* is a trigonometric polynomial of degree *q*, $G^{*}$ is also a trigonometric polynomial of degree *q*, where $G^{*}\left(\omega \right):={\left(G\left(\omega \right)\right)}^{*}$ for all ω∈R. Applying [6], Lemma 4.2, and Equation (4) yields:

$$\begin{array}{cc} ∥T_{n}\left(F\right)T_{n}\left(G\right)-T_{n}{\left(FG\right)∥}_{F}= & {∥{\left(T_{n}\left(F\right)T_{n}\left(G\right)-T_{n}\left(FG\right)\right)}^{*}∥}_{F}\\ = & {∥{\left(T_{n}\left(G\right)\right)}^{*}{\left(T_{n}\left(F\right)\right)}^{*}-{\left(T_{n}\left(FG\right)\right)}^{*}∥}_{F}\\ = & {∥T_{n}\left(G^{*}\right)T_{n}\left(F^{*}\right)-T_{n}\left(G^{*}F^{*}\right)∥}_{F}\\ \leq & \sqrt{q\left(q+1\right)\left(\frac{1}{2\pi }\int_{0}^{2\pi }{∥{\left(G\left(\omega \right)\right)}^{*}∥}_{F}^{2}d\omega \right)\left(\frac{1}{2\pi }\int_{0}^{2\pi }{∥{\left(F\left(\omega \right)\right)}^{*}∥}_{F}^{2}d\omega \right)}, \end{array}$$

and thus, Equation (6) is proven. (3) Fix n≥max{2p,2q}. As $F_{r}=0_{M\times N}$ with |r|>p and $G_{s}=0_{N\times K}$ with |s|>q, from Lemma 1, we obtain:

$$\begin{array}{cc} {\left[T_{n}\left(F\right)T_{n}\left(G\right)-T_{n}\left(FG\right)\right]}_{j,k} & =-\lim_{H\to \infty }\left(\sum_{h=-H}^{0}F_{j-h}G_{h-k}+\sum_{h=n+1}^{H}F_{j-h}G_{h-k}\right)\\ \; & =-\sum_{h=\max\{j-p,k-q\}}^{0}F_{j-h}G_{h-k}-\sum_{h=n+1}^{\min\{j+p,k+q\}}F_{j-h}G_{h-k}\\ \; & =\left\{\begin{array}{cc} -\sum_{h=\max\{j-p,k-q\}}^{0}F_{j-h}G_{h-k} & \mathrm{if}\;j\leq p\;\mathrm{and}\;k\leq q,\\ 0_{M\times K} & \mathrm{if}\;j\leq p\;\mathrm{and}\;k\geq q+1,\\ 0_{M\times K} & \mathrm{if}\;p+1\leq j\leq n-p,\\ 0_{M\times K} & \mathrm{if}\;j\geq n-p+1\;\mathrm{and}\;k\leq n-q,\\ -\sum_{h=n+1}^{\min\{j+p,k+q\}}F_{j-h}G_{h-k} & \mathrm{if}\;j\geq n-p+1\;\mathrm{and}\;k\geq n-q+1, \end{array}\right. \end{array}$$

for all j,k∈{1,…,n}. Observe that:

$${\left[T_{n}\left(F\right)T_{n}\left(G\right)-T_{n}\left(FG\right)\right]}_{n-p+j,n-q+k}=-\sum_{h=n+1}^{\min\{n+j,n+k\}}F_{n-p+j-h}G_{h-n+q-k}=-\sum_{l=1}^{\min\{j,k\}}F_{-p+j-l}G_{l+q-k}$$

for all j∈{1,…,p} and k∈{1,…,q}. □

## Appendix C. Proof of Lemma 3

**Proof.** (1) Since $F:R\to C^{N\times N}$ is continuous and 2π-periodic, $F^{-1}:R\to C^{N\times N}$ is also continuous and 2π-periodic, where $F^{-1}\left(\omega \right):={\left(F\left(\omega \right)\right)}^{-1}$ for all ω∈R. As:

$$\begin{array}{cc} ∥{\left(T_{n}\left(F\right)\right)}^{-1}-T_{n}\left(F^{-1}\right){∥}_{F}= & ∥{\left(T_{n}\left(F\right)\right)}^{-1}\left(I_{nN}-T_{n}\left(F\right)T_{n}\left(F^{-1}\right)\right){∥}_{F}\\ \leq & ∥{\left(T_{n}\left(F\right)\right)}^{-1}{∥}_{2}{∥I_{nN}-T_{n}\left(F\right)T_{n}\left(F^{-1}\right)∥}_{F}\\ = & ∥{\left(T_{n}\left(F\right)\right)}^{-1}{∥}_{2}{∥T_{n}\left(I_{N}\right)-T_{n}\left(F\right)T_{n}\left(F^{-1}\right)∥}_{F}\\ = & ∥{\left(T_{n}\left(F\right)\right)}^{-1}{∥}_{2}{∥T_{n}\left(F\right)T_{n}\left(F^{-1}\right)-T_{n}\left(FF^{-1}\right)∥}_{F} \end{array}$$

for all n∈N, Equation (4) proves Assertion 1 of Lemma 3. (2) Since F(ω) is positive definite for all ω∈R (or equivalently, F(ω) is Hermitian and $\lambda_{N}\left(F\left(\omega \right)\right)>0$ for all ω∈R), F(ω) is invertible for all ω∈R (or equivalently, $\det\left(F\left(\omega \right)\right)=\prod_{k=1}^{N}\lambda_{k}\left(F\left(\omega \right)\right)$ is non-zero for all ω∈R), $T_{n}\left(F\right)$ is Hermitian, and $\lambda_{nN}\left(T_{n}\left(F\right)\right)\geq \inf F>0$ for all n∈N (see Equation (1)). As $T_{n}\left(F\right)$ is positive definite for all n∈N, ${\left(T_{n}\left(F\right)\right)}^{-1}$ is also positive definite for all n∈N. Therefore,

$$∥{\left(T_{n}\left(F\right)\right)}^{-1}{∥}_{2}=\lambda_{1}\left({\left(T_{n}\left(F\right)\right)}^{-1}\right)=\frac{1}{\lambda_{nN}\left(T_{n}\left(F\right)\right)}\leq \frac{1}{\inf F}$$

for all n∈N. Assertion 2 of Lemma 3 can now be obtained from Assertion 1 of Lemma 3. □

## Appendix D. Proof of Lemma 4

**Proof.** Consider $A_{n},B_{n}\in C^{nM\times nN}$. As $V_{n}⊗I_{m}$ is unitary, ${\left(V_{n}⊗I_{m}\right)}^{*}$ is also unitary for all m∈N. Consequently, since the Frobenius norm is unitarily invariant, we have

$$\begin{array}{cc} ∥C_{A_{n}}-C_{B_{n}}{∥}_{F} & ={∥\left(V_{n}⊗I_{M}\right){\mathrm{diag}}_{1\leq k\leq n}\left({\left[{\left(V_{n}⊗I_{M}\right)}^{*}\left(A_{n}-B_{n}\right)\left(V_{n}⊗I_{N}\right)\right]}_{k,k}\right){\left(V_{n}⊗I_{N}\right)}^{*}∥}_{F}\\ \; & ={∥{\mathrm{diag}}_{1\leq k\leq n}\left({\left[{\left(V_{n}⊗I_{M}\right)}^{*}\left(A_{n}-B_{n}\right)\left(V_{n}⊗I_{N}\right)\right]}_{k,k}\right)∥}_{F}\\ \; & \leq {∥{\left(V_{n}⊗I_{M}\right)}^{*}\left(A_{n}-B_{n}\right)\left(V_{n}⊗I_{N}\right)∥}_{F}={∥A_{n}-B_{n}∥}_{F} \end{array}$$

and:

$$\begin{array}{cc} ∥A_{n}-C_{A_{n}}{∥}_{F} & \leq ∥A_{n}-C_{B_{n}}{∥}_{F}+{∥C_{B_{n}}-C_{A_{n}}∥}_{F}\\ \; & \leq ∥A_{n}-B_{n}{∥}_{F}+∥B_{n}-C_{B_{n}}{∥}_{F}+∥C_{A_{n}}-C_{B_{n}}{∥}_{F}\leq 2∥A_{n}-B_{n}{∥}_{F}+{∥B_{n}-C_{B_{n}}∥}_{F}. \end{array}$$

If $B_{n}$ is an n×n block circulant matrix with M×N blocks, then (see, e.g., [6], Lemma 5.1, or [12], Lemma 3) there exist $\Lambda_{1},\ldots ,\Lambda_{n}\in C^{M\times N}$ such that:

$$B_{n}=\left(V_{n}⊗I_{M}\right)\mathrm{diag}\left(\Lambda_{1},\ldots ,\Lambda_{n}\right){\left(V_{n}⊗I_{N}\right)}^{*}.$$

Therefore,

$$\begin{array}{cc} C_{B_{n}} & =\left(V_{n}⊗I_{M}\right){\mathrm{diag}}_{1\leq k\leq n}\left({\left[{\left(V_{n}⊗I_{M}\right)}^{*}B_{n}\left(V_{n}⊗I_{N}\right)\right]}_{k,k}\right){\left(V_{n}⊗I_{N}\right)}^{*}\\ \; & =\left(V_{n}⊗I_{M}\right){\mathrm{diag}}_{1\leq k\leq n}\left({\left[\mathrm{diag}\left(\Lambda_{1},\ldots ,\Lambda_{n}\right)\right]}_{k,k}\right){\left(V_{n}⊗I_{N}\right)}^{*}\\ \; & =\left(V_{n}⊗I_{M}\right){\mathrm{diag}}_{1\leq k\leq n}\left(\Lambda_{k}\right){\left(V_{n}⊗I_{N}\right)}^{*}=B_{n}, \end{array}$$

and combining Equations (9) and (10), we obtain Equation (11). □

## Appendix E. Proof of Lemma 5

**Proof.** Fix n>2p. From [13], p. 5, we obtain:

$$\begin{array}{cc} {\left[{\hat{C}}_{n}\left(F\right)\right]}_{j,1} & =\left\{\begin{array}{cc} F_{0} & \mathrm{if}\;j=1,\\ \left(1-\frac{j-1}{n}\right)F_{j-1}+\frac{j-1}{n}F_{j-1-n} & \mathrm{if}\;j\in \{2,\ldots ,n\}, \end{array}\right.\\ \; & =\left\{\begin{array}{cc} F_{0} & \mathrm{if}\;j=1,\\ \left(1-\frac{j-1}{n}\right)F_{j-1} & \mathrm{if}\;j\in \{2,\ldots ,p+1\},\\ 0_{M\times N} & \mathrm{if}\;j\in \{p+2,\ldots ,n-p\},\\ \frac{j-1}{n}F_{j-1-n} & \mathrm{if}\;j\in \{n-p+1,\ldots ,n\}. \end{array}\right. \end{array}$$

Hence, the Frobenius norm of the n×n block Toeplitz matrix with M×N blocks $T_{n}\left(F\right)-{\hat{C}}_{n}\left(F\right)$ is given by:

(A1) $$\begin{array}{cc} ∥T_{n}\left(F\right)-{\hat{C}}_{n}{\left(F\right)∥}_{F}^{2}= & \sum_{j,k=1}^{n}{∥{\left[T_{n}\left(F\right)-{\hat{C}}_{n}\left(F\right)\right]}_{j,k}∥}_{F}^{2}\\ = & \sum_{j=1}^{n}\left(n-j+1\right)∥{\left[T_{n}\left(F\right)-{\hat{C}}_{n}\left(F\right)\right]}_{j,1}{∥}_{F}^{2}+\sum_{k=2}^{n}\left(n-k+1\right){∥{\left[T_{n}\left(F\right)-{\hat{C}}_{n}\left(F\right)\right]}_{1,k}∥}_{F}^{2}\\ = & \sum_{j=1}^{n}\left(n-j+1\right)∥{\left[T_{n}\left(F\right)\right]}_{j,1}\;-\;{\left[{\hat{C}}_{n}\left(F\right)\right]}_{j,1}{∥}_{F}^{2}+\sum_{k=2}^{n}\left(n-k+1\right){∥{\left[T_{n}\left(F\right)\right]}_{1,k}\;-\;{\left[{\hat{C}}_{n}\left(F\right)\right]}_{1,k}∥}_{F}^{2}\\ = & \sum_{j=1}^{n}\left(n-j+1\right)∥F_{j-1}-{\left[{\hat{C}}_{n}\left(F\right)\right]}_{j,1}{∥}_{F}^{2}+\sum_{k=2}^{n}\left(n-k+1\right){∥F_{1-k}-{\left[{\hat{C}}_{n}\left(F\right)\right]}_{n+2-k,1}∥}_{F}^{2}\\ = & \sum_{j=2}^{n}\left(n-j+1\right)∥F_{j-1}-{\left[{\hat{C}}_{n}\left(F\right)\right]}_{j,1}{∥}_{F}^{2}+\sum_{h=2}^{n}\left(h-1\right){∥F_{h-1-n}-{\left[{\hat{C}}_{n}\left(F\right)\right]}_{h,1}∥}_{F}^{2}\\ = & \sum_{j=2}^{n}\left(\left(n-j+1\right)∥F_{j-1}-{\left[{\hat{C}}_{n}\left(F\right)\right]}_{j,1}{∥}_{F}^{2}+\left(j-1\right){∥F_{j-1-n}-{\left[{\hat{C}}_{n}\left(F\right)\right]}_{j,1}∥}_{F}^{2}\right)\\ = & \sum_{j=2}^{p+1}\left(\left(n-j+1\right)∥F_{j-1}-{\left[{\hat{C}}_{n}\left(F\right)\right]}_{j,1}{∥}_{F}^{2}+\left(j-1\right){∥F_{j-1-n}-{\left[{\hat{C}}_{n}\left(F\right)\right]}_{j,1}∥}_{F}^{2}\right)\\ & +\sum_{j=n-p+1}^{n}\left(\left(n-j+1\right)∥F_{j-1}-{\left[{\hat{C}}_{n}\left(F\right)\right]}_{j,1}{∥}_{F}^{2}+\left(j-1\right){∥F_{j-1-n}-{\left[{\hat{C}}_{n}\left(F\right)\right]}_{j,1}∥}_{F}^{2}\right)\\ = & \sum_{j=2}^{p+1}\left(\left(n-j+1\right){∥F_{j-1}-\left(1-\frac{j-1}{n}\right)F_{j-1}∥}_{F}^{2}+\left(j-1\right){∥-\left(1-\frac{j-1}{n}\right)F_{j-1}∥}_{F}^{2}\right)\\ & +\sum_{j=n-p+1}^{n}\left(\left(n-j+1\right){∥-\frac{j-1}{n}F_{j-1-n}∥}_{F}^{2}+\left(j-1\right){∥F_{j-1-n}-\frac{j-1}{n}F_{j-1-n}∥}_{F}^{2}\right)\\ = & \sum_{j=2}^{p+1}\left(\left(n-j+1\right){\left(\frac{j-1}{n}\right)}^{2}{∥F_{j-1}∥}_{F}^{2}+\left(j-1\right){\left(\frac{n-j+1}{n}\right)}^{2}{∥F_{j-1}∥}_{F}^{2}\right)\\ & +\sum_{j=n-p+1}^{n}\;\left(\left(n-j+1\right){\left(\frac{j-1}{n}\right)}^{2}{∥F_{j-1-n}∥}_{F}^{2}+\left(j-1\right){\left(\frac{n-j+1}{n}\right)}^{2}{∥F_{j-1-n}∥}_{F}^{2}\right)\\ = & \sum_{j=2}^{p+1}\frac{\left(n-j+1)(j-1\right)}{n}∥F_{j-1}{∥}_{F}^{2}+\sum_{j=n-p+1}^{n}\frac{\left(n-j+1)(j-1\right)}{n}{∥F_{j-1-n}∥}_{F}^{2}\\ = & \sum_{k=1}^{p}k\frac{n-k}{n}∥F_{k}{∥}_{F}^{2}+\sum_{h=1}^{p}h\frac{n-h}{n}{∥F_{-h}∥}_{F}^{2}\\ = & \sum_{k=1}^{p}k\frac{n-k}{n}\left(∥F_{k}{∥}_{F}^{2}+{∥F_{-k}∥}_{F}^{2}\right). \end{array}$$

Applying [6], Lemma 5.4, we have:

$$\begin{array}{cc} {\left[C_{n}\left(F\right)\right]}_{j,1} & =\left\{\begin{array}{cc} F_{0} & \mathrm{if}\;j=1,\\ F_{j-1} & \mathrm{if}\;j\in \{2,\ldots ,p+1\},\\ 0_{M\times N} & \mathrm{if}\;j\in \{p+2,\ldots ,n-p\},\\ F_{j-1-n} & \mathrm{if}\;j\in \{n-p+1,\ldots ,n\}. \end{array}\right. \end{array}$$

Consequently, the Frobenius norm of the n×n block Toeplitz matrix with M×N blocks $T_{n}\left(F\right)-C_{n}\left(F\right)$ is given by:

(A2) $$\begin{array}{cc} ∥T_{n}\left(F\right)-C_{n}{\left(F\right)∥}_{F}^{2}= & \sum_{j=2}^{p+1}\left(\left(n-j+1\right)∥F_{j-1}-{\left[C_{n}\left(F\right)\right]}_{j,1}{∥}_{F}^{2}+\left(j-1\right){∥F_{j-1-n}-{\left[C_{n}\left(F\right)\right]}_{j,1}∥}_{F}^{2}\right)\\ & +\sum_{j=n-p+1}^{n}\left(\left(n-j+1\right)∥F_{j-1}-{\left[C_{n}\left(F\right)\right]}_{j,1}{∥}_{F}^{2}+\left(j-1\right){∥F_{j-1-n}-{\left[C_{n}\left(F\right)\right]}_{j,1}∥}_{F}^{2}\right)\\ = & \sum_{j=2}^{p+1}\left(j-1\right)∥-F_{j-1}{∥}_{F}^{2}+\sum_{j=n-p+1}^{n}\left(n-j+1\right){∥-F_{j-1-n}∥}_{F}^{2}\\ = & \sum_{k=1}^{p}k∥F_{k}{∥}_{F}^{2}+\sum_{h=1}^{p}h{∥F_{-h}∥}_{F}^{2}\\ = & \sum_{k=1}^{p}k\left(∥F_{k}{∥}_{F}^{2}+{∥F_{-k}∥}_{F}^{2}\right). \end{array}$$

Equations (A1) and (A2) prove Lemma 5. □

## Appendix F. Proof of Lemma 7

**Proof.** From Equation (12), we obtain:

$$x_{n:1}=T_{n}\left(G\right)w_{n:1}\;\forall n\in N,$$

with $G\left(\omega \right)=I_{N}+\sum_{k=1}^{q}e^{-k\omega i}G_{-k}$ for all ω∈R. Therefore, applying [6], Lemma 4.2, yields:

(A3) $$\left\{E\left(x_{n:1}x_{n:1}^{*}\right)\right\}=\left\{T_{n}\left(G\right)E\left(w_{n:1}w_{n:1}^{*}\right){\left(T_{n}\left(G\right)\right)}^{*}\right\}=\left\{T_{n}\left(G\right)T_{n}\left(\Lambda \right)T_{n}\left(G^{*}\right)\right\}=\left\{T_{n}\left(G\Lambda \right)T_{n}\left(G^{*}\right)\right\}.$$

Hence, using Equation (9), we have:

$$\begin{array}{cc} {∥E\left(x_{n:1}x_{n:1}^{*}\right)-C_{E\left(x_{n:1}x_{n:1}^{*}\right)}∥}_{F} & \leq 2∥E\left(x_{n:1}x_{n:1}^{*}\right)-T_{n}\left(G\Lambda G^{*}\right){∥}_{F}+{∥T_{n}\left(G\Lambda G^{*}\right)-C_{T_{n}\left(G\Lambda G^{*}\right)}∥}_{F}\\ \; & =2∥T_{n}\left(G\Lambda \right)T_{n}\left(G^{*}\right)-T_{n}\left(G\Lambda G^{*}\right){∥}_{F}+{∥T_{n}\left(G\Lambda G^{*}\right)-{\hat{C}}_{n}\left(G\Lambda G^{*}\right)∥}_{F} \end{array}$$

for all n∈N. Thus, to finish the proof, we only need to show that $\left\{∥T_{n}\left(G\Lambda \right)T_{n}\left(G^{*}\right)-T_{n}\left(G\Lambda G^{*}\right){∥}_{F}\right\}$ and $\left\{{∥T_{n}\left(G\Lambda G^{*}\right)-{\hat{C}}_{n}\left(G\Lambda G^{*}\right)∥}_{F}\right\}$ are bounded. As GΛ and $G^{*}$ are trigonometric polynomials, from Equation (7), we obtain that $\left\{∥T_{n}\left(G\Lambda \right)T_{n}\left(G^{*}\right)-T_{n}\left(G\Lambda G^{*}\right){∥}_{F}\right\}$ is bounded. Since $G\Lambda G^{*}$ is a trigonometric polynomial, applying Lemma 5, we conclude that $\left\{{∥T_{n}\left(G\Lambda G^{*}\right)-{\hat{C}}_{n}\left(G\Lambda G^{*}\right)∥}_{F}\right\}$ is bounded. □

## Appendix G. Proof of Lemma 8

**Proof.** As Λ is positive definite, if ω∈R and $v\in C^{N\times 1}$, then:

$$v^{*}{\left(F\left(\omega \right)\right)}^{-1}\Lambda {\left({\left(F\left(\omega \right)\right)}^{-1}\right)}^{*}v\;=\;{\left({\left({\left(F\left(\omega \right)\right)}^{-1}\right)}^{*}v\right)}^{*}\Lambda {\left({\left(F\left(\omega \right)\right)}^{-1}\right)}^{*}v\;=\;{\left({\left({\left(F\left(\omega \right)\right)}^{*}\right)}^{-1}v\right)}^{*}\Lambda {\left({\left(F\left(\omega \right)\right)}^{*}\right)}^{-1}v>0$$

whenever ${\left({\left(F\left(\omega \right)\right)}^{*}\right)}^{-1}v\neq 0_{N\times 1}$, or equivalently, $v\neq 0_{N\times 1}$. Since ${\left(F\left(\omega \right)\right)}^{-1}\Lambda {\left({\left(F\left(\omega \right)\right)}^{-1}\right)}^{*}$ is positive definite for all ω∈R, we have:

$$∥{\left(T_{n}\left(F^{-1}\Lambda {\left(F^{-1}\right)}^{*}\right)\right)}^{-1}{∥}_{2}\leq \frac{1}{\inf(F^{-1}\Lambda {\left(F^{-1}\right)}^{*})}\;\forall n\in N.$$

From Equation (13), we obtain:

$$w_{n:1}=T_{n}\left(F\right)x_{n:1}\;\forall n\in N.$$

Consequently,

$$\left\{T_{n}\left(\Lambda \right)\right\}=\left\{E\left(w_{n:1}w_{n:1}^{*}\right)\right\}=\left\{T_{n}\left(F\right)E\left(x_{n:1}x_{n:1}^{*}\right){\left(T_{n}\left(F\right)\right)}^{*}\right\}.$$

Therefore, as $\det(T_{n}\left(F\right))=1$ for all n∈N, we have:

(A4) $$\left\{E\left(x_{n:1}x_{n:1}^{*}\right)\right\}=\left\{{\left(T_{n}\left(F\right)\right)}^{-1}T_{n}\left(\Lambda \right){\left({\left(T_{n}\left(F\right)\right)}^{*}\right)}^{-1}\right\}.$$

Hence, applying Equation (11) and [6], Lemma 4.2, yields:

$$\begin{array}{cc} \; & \;\;\;\;\;\;\;\;\;{∥E\left(x_{n:1}x_{n:1}^{*}\right)-C_{E\left(x_{n:1}x_{n:1}^{*}\right)}∥}_{F}\\ \leq & 2∥E\left(x_{n:1}x_{n:1}^{*}\right)-C_{n}\left(F^{-1}\Lambda {\left(F^{-1}\right)}^{*}\right){∥}_{F}\\ \leq & 2\left(\;∥{\left(T_{n}\left(F\right)\right)}^{-1}T_{n}\left(\Lambda \right){\left({\left(T_{n}\left(F\right)\right)}^{*}\right)}^{-1}\;-\;T_{n}\left(F^{-1}\Lambda {\left(F^{-1}\right)}^{*}\right){∥}_{F}\;+\;{∥T_{n}\left(F^{-1}\Lambda {\left(F^{-1}\right)}^{*}\right)\;-\;C_{n}\left(F^{-1}\Lambda {\left(F^{-1}\right)}^{*}\right)∥}_{F}\;\right)\\ \leq & 2\left(∥{\left(T_{n}\left(F\right)\right)}^{-1}T_{n}\left(\Lambda \right){\left({\left(T_{n}\left(F\right)\right)}^{*}\right)}^{-1}{∥}_{2}{∥I_{nN}-{\left(T_{n}\left(F\right)\right)}^{*}{\left(T_{n}\left(\Lambda \right)\right)}^{-1}T_{n}\left(F\right)T_{n}\left(F^{-1}\Lambda {\left(F^{-1}\right)}^{*}\right)∥}_{F}\right.\\ \; & +\left.∥T_{n}\left(F^{-1}\Lambda {\left(F^{-1}\right)}^{*}\right){∥}_{2}{∥I_{nN}-{\left(T_{n}\left(F^{-1}\Lambda {\left(F^{-1}\right)}^{*}\right)\right)}^{-1}C_{n}\left(F^{-1}\Lambda {\left(F^{-1}\right)}^{*}\right)∥}_{F}\right)\\ \leq & 2\left(∥{\left(T_{n}\left(F\right)\right)}^{-1}{∥}_{2}∥T_{n}{\left(\Lambda \right)∥}_{2}∥{\left({\left(T_{n}\left(F\right)\right)}^{*}\right)}^{-1}{∥}_{2}{∥I_{nN}-T_{n}\left(F^{*}\right)T_{n}\left(\Lambda^{-1}\right)T_{n}\left(F\right)T_{n}\left(F^{-1}\Lambda {\left(F^{-1}\right)}^{*}\right)∥}_{F}\right.\\ \; & +\left.∥T_{n}\left(F^{-1}\Lambda {\left(F^{-1}\right)}^{*}\right){∥}_{2}∥{\left(C_{n}\left(F^{-1}\Lambda {\left(F^{-1}\right)}^{*}\right)\right)}^{-1}-{\left(T_{n}\left(F^{-1}\Lambda {\left(F^{-1}\right)}^{*}\right)\right)}^{-1}{∥}_{F}{∥C_{n}\left(F^{-1}\Lambda {\left(F^{-1}\right)}^{*}\right)∥}_{2}\right)\\ = & 2\left(∥{\left(T_{n}\left(F\right)\right)}^{-1}{∥}_{2}\lambda_{1}\left(\Lambda \right)∥{\left({\left(T_{n}\left(F\right)\right)}^{-1}\right)}^{*}{∥}_{2}{∥I_{nN}-T_{n}\left(F^{*}\right)T_{n}\left(\Lambda^{-1}F\right)T_{n}\left(F^{-1}\Lambda {\left(F^{-1}\right)}^{*}\right)∥}_{F}\right.\\ \; & +\left.∥T_{n}\left(F^{-1}\Lambda {\left(F^{-1}\right)}^{*}\right){∥}_{2}∥C_{n}\left({\left(F^{-1}\Lambda {\left(F^{-1}\right)}^{*}\right)}^{-1}\right)-{\left(T_{n}\left(F^{-1}\Lambda {\left(F^{-1}\right)}^{*}\right)\right)}^{-1}{∥}_{F}{∥C_{n}\left(F^{-1}\Lambda {\left(F^{-1}\right)}^{*}\right)∥}_{2}\right)\\ \leq & 2∥T_{n}\left(F^{-1}\Lambda {\left(F^{-1}\right)}^{*}\right){∥}_{2}\left(∥{\left(T_{n}\left(F\right)\right)}^{-1}{∥}_{2}^{2}\lambda_{1}\left(\Lambda \right){∥{\left(T_{n}\left(F^{-1}\Lambda {\left(F^{-1}\right)}^{*}\right)\right)}^{-1}-T_{n}\left(F^{*}\right)T_{n}\left(\Lambda^{-1}F\right)∥}_{F}\right.\\ \; & +\left.∥C_{n}\left(F^{-1}\Lambda {\left(F^{-1}\right)}^{*}\right){∥}_{2}{∥C_{n}\left({\left(F^{-1}\Lambda {\left(F^{*}\right)}^{-1}\right)}^{-1}\right)-{\left(T_{n}\left(F^{-1}\Lambda {\left(F^{-1}\right)}^{*}\right)\right)}^{-1}∥}_{F}\right)\\ \leq & 2∥T_{n}\left(F^{-1}\Lambda {\left(F^{-1}\right)}^{*}\right){∥}_{2}\left(∥{\left(T_{n}\left(F\right)\right)}^{-1}{∥}_{2}^{2}\lambda_{1}\left(\Lambda \right)\left(∥{\left(T_{n}\left(F^{-1}\Lambda {\left(F^{-1}\right)}^{*}\right)\right)}^{-1}-T_{n}\left(F^{*}\Lambda^{-1}F\right){∥}_{F}\right.\right.\\ \; & +\left.∥T_{n}\left(F^{*}\Lambda^{-1}F\right)-T_{n}\left(F^{*}\right)T_{n}\left(\Lambda^{-1}F\right){∥}_{F}\right)+{∥C_{n}\left(F^{-1}\Lambda {\left(F^{-1}\right)}^{*}\right)∥}_{2}\left(∥C_{n}\left(F^{*}\Lambda^{-1}F\right)-T_{n}\left(F^{*}\Lambda^{-1}F\right){∥}_{F}\right.\\ \; & +\left.\left.∥T_{n}\left(F^{*}\Lambda^{-1}F\right)-{\left(T_{n}\left(F^{-1}\Lambda {\left(F^{-1}\right)}^{*}\right)\right)}^{-1}{∥}_{F}\right)\right)\\ = & 2∥T_{n}\left(F^{-1}\Lambda {\left(F^{-1}\right)}^{*}\right){∥}_{2}\left(∥{\left(T_{n}\left(F\right)\right)}^{-1}{∥}_{2}^{2}\lambda_{1}\left(\Lambda \right){∥T_{n}\left(F^{*}\right)T_{n}\left(\Lambda^{-1}F\right)-T_{n}\left(F^{*}\Lambda^{-1}F\right)∥}_{F}\right.\\ \; & +\left(∥{\left(T_{n}\left(F\right)\right)}^{-1}{∥}_{2}^{2}\lambda_{1}\left(\Lambda \right)+{∥C_{n}\left(F^{-1}\Lambda {\left(F^{-1}\right)}^{*}\right)∥}_{2}\right){∥{\left(T_{n}\left(F^{-1}\Lambda {\left(F^{-1}\right)}^{*}\right)\right)}^{-1}-T_{n}\left(F^{*}\Lambda^{-1}F\right)∥}_{F}\\ \; & +\left.∥C_{n}\left(F^{-1}\Lambda {\left(F^{-1}\right)}^{*}\right){∥}_{2}{∥T_{n}\left(F^{*}\Lambda^{-1}F\right)-C_{n}\left(F^{*}\Lambda^{-1}F\right)∥}_{F}\right)\\ \leq & 2∥T_{n}\left(F^{-1}\Lambda {\left(F^{-1}\right)}^{*}\right){∥}_{2}\left(∥{\left(T_{n}\left(F\right)\right)}^{-1}{∥}_{2}^{2}\lambda_{1}\left(\Lambda \right){∥T_{n}\left(F^{*}\right)T_{n}\left(\Lambda^{-1}F\right)-T_{n}\left(F^{*}\Lambda^{-1}F\right)∥}_{F}\right.\\ \; & +\left(∥{\left(T_{n}\left(F\right)\right)}^{-1}{∥}_{2}^{2}\lambda_{1}\left(\Lambda \right)+{∥C_{n}\left(F^{-1}\Lambda {\left(F^{-1}\right)}^{*}\right)∥}_{2}\right)∥{\left(T_{n}\left(F^{-1}\Lambda {\left(F^{-1}\right)}^{*}\right)\right)}^{-1}{∥}_{2}{∥T_{n}\left(F^{*}\Lambda^{-1}F\right)∥}_{2}\\ \; & \;\;\;\;\times ∥{\left(T_{n}\left(F^{*}\Lambda^{-1}F\right)\right)}^{-1}-T_{n}\left(F^{-1}\Lambda {\left(F^{-1}\right)}^{*}\right){∥}_{F}\\ \; & +\left.∥C_{n}\left(F^{-1}\Lambda {\left(F^{-1}\right)}^{*}\right){∥}_{2}{∥T_{n}\left(F^{*}\Lambda^{-1}F\right)-C_{n}\left(F^{*}\Lambda^{-1}F\right)∥}_{F}\right)\\ = & 2∥T_{n}\left(F^{-1}\Lambda {\left(F^{-1}\right)}^{*}\right){∥}_{2}\left(∥{\left(T_{n}\left(F\right)\right)}^{-1}{∥}_{2}^{2}\lambda_{1}\left(\Lambda \right){∥T_{n}\left(F^{*}\right)T_{n}\left(\Lambda^{-1}F\right)-T_{n}\left(F^{*}\Lambda^{-1}F\right)∥}_{F}\right.\\ \; & +\left(∥{\left(T_{n}\left(F\right)\right)}^{-1}{∥}_{2}^{2}\lambda_{1}\left(\Lambda \right)+{∥C_{n}\left(F^{-1}\Lambda {\left(F^{-1}\right)}^{*}\right)∥}_{2}\right)∥{\left(T_{n}\left(F^{-1}\Lambda {\left(F^{-1}\right)}^{*}\right)\right)}^{-1}{∥}_{2}{∥T_{n}\left(F^{*}\Lambda^{-1}F\right)∥}_{2}\\ \; & \;\;\;\;\times ∥{\left(T_{n}\left(F^{*}\Lambda^{-1}F\right)\right)}^{-1}-T_{n}\left({\left(F^{*}\Lambda^{-1}F\right)}^{-1}\right){∥}_{F}\\ \; & +\left.∥C_{n}\left(F^{-1}\Lambda {\left(F^{-1}\right)}^{*}\right){∥}_{2}{∥T_{n}\left(F^{*}\Lambda^{-1}F\right)-C_{n}\left(F^{*}\Lambda^{-1}F\right)∥}_{F}\right) \end{array}$$

for all n∈N. Thus, to finish the proof, we only need to show that $\left\{∥T_{n}\left(F^{-1}\Lambda {\left(F^{-1}\right)}^{*}\right){∥}_{2}\right\}$, $\left\{∥T_{n}\left(F^{*}\right)T_{n}\left(\Lambda^{-1}F\right)-T_{n}\left(F^{*}\Lambda^{-1}F\right){∥}_{F}\right\}$, $\left\{∥C_{n}\left(F^{-1}\Lambda {\left(F^{-1}\right)}^{*}\right){∥}_{2}\right\}$, $\left\{∥T_{n}\left(F^{*}\Lambda^{-1}F\right){∥}_{2}\right\}$, $\left\{∥{\left(T_{n}\left(F^{*}\Lambda^{-1}F\right)\right)}^{-1}-T_{n}\left({\left(F^{*}\Lambda^{-1}F\right)}^{-1}\right){∥}_{F}\right\}$, and $\left\{∥T_{n}\left(F^{*}\Lambda^{-1}F\right)-C_{n}\left(F^{*}\Lambda^{-1}F\right){∥}_{F}\right\}$ are bounded. From [6], Theorem 4.3, we obtain that $\left\{∥T_{n}\left(F^{-1}\Lambda {\left(F^{-1}\right)}^{*}\right){∥}_{2}\right\}$ and $\left\{∥T_{n}\left(F^{*}\Lambda^{-1}F\right){∥}_{2}\right\}$ are bounded. Applying [6], Lemma 5.2, we have that $\left\{∥C_{n}\left(F^{-1}\Lambda {\left(F^{-1}\right)}^{*}\right){∥}_{2}\right\}$ is bounded. Since $F^{*}$ and $\Lambda^{-1}F$ are trigonometric polynomials, from Equation (7), we obtain that $\left\{∥T_{n}\left(F^{*}\right)T_{n}\left(\Lambda^{-1}F\right)-T_{n}\left(F^{*}\Lambda^{-1}F\right){∥}_{F}\right\}$ is bounded. As $F^{*}\Lambda^{-1}F$ is a trigonometric polynomial, applying Lemma 5, we have that $\left\{∥T_{n}\left(F^{*}\Lambda^{-1}F\right)-C_{n}\left(F^{*}\Lambda^{-1}F\right){∥}_{F}\right\}$ is bounded. Since ${\left(F\left(\omega \right)\right)}^{-1}\Lambda {\left({\left(F\left(\omega \right)\right)}^{-1}\right)}^{*}={\left(F\left(\omega \right)\right)}^{-1}\Lambda {\left({\left(F\left(\omega \right)\right)}^{*}\right)}^{-1}$ is positive definite for all ω∈R, ${\left({\left(F\left(\omega \right)\right)}^{-1}\Lambda {\left({\left(F\left(\omega \right)\right)}^{*}\right)}^{-1}\right)}^{-1}={\left(F\left(\omega \right)\right)}^{*}\Lambda^{-1}F\left(\omega \right)$ is also positive definite for all ω∈R, and consequently, from Equation (8), we conclude that $\left\{∥{\left(T_{n}\left(F^{*}\Lambda^{-1}F\right)\right)}^{-1}-T_{n}\left({\left(F^{*}\Lambda^{-1}F\right)}^{-1}\right){∥}_{F}\right\}$ is bounded. □

## Appendix H. A Statistical Signal Processing Application on Filtering WSS Vector Processes

Consider a zero-mean WSS *M*-dimensional vector process $\left\{x_{n}\right\}$. Let *Y* be the PSD of a zero-mean WSS *N*-dimensional vector process $\left\{y_{n}\right\}$ with infY>0. Assume that those two processes are jointly WSS with joint PSD *Z*, that is $Z:R\to C^{M\times N}$ is a continuous 2π-periodic function satisfying that $\left\{E\left(x_{n:1}y_{n:1}^{*}\right)\right\}=\left\{T_{n}\left(Z\right)\right\}$.

For every n∈N, if ${\tilde{x}}_{n:1}$ is an estimation of $x_{n:1}$ from $y_{n:1}$ of the form:

(A5) $${\tilde{x}}_{n:1}=Wy_{n:1}$$

with $W\in C^{nM\times nN}$, the MSE per sample is:

$$\frac{\mathrm{MSE}(W)}{n}=\frac{E\left(∥x_{n:1}-{\tilde{x}}_{n:1}{∥}_{2}^{2}\right)}{n},$$

and the minimum MSE (MMSE) is given by $\mathrm{MMSE}=\mathrm{MSE}(W_{0})$, where $W_{0}$ is the Wiener filter, i.e.,

$$W_{0}=E\left(x_{n:1}y_{n:1}^{*}\right){\left(E\left(y_{n:1}y_{n:1}^{*}\right)\right)}^{-1}=T_{n}\left(Z\right){\left(T_{n}\left(Y\right)\right)}^{-1}.$$

In [13], Equation (6), it was shown that there is no difference in the MSE per sample for large enough *n* if we substitute the optimal filter $W_{0}$ by $W_{C}$, where $W_{C}={\hat{C}}_{n}\left(Z\right){\left({\hat{C}}_{n}\left(Y\right)\right)}^{-1}$, that is,

$$\lim_{n\to \infty }\left(\frac{\mathrm{MSE}(W_{C})}{n}-\frac{\mathrm{MMSE}}{n}\right)=0.$$

Obviously, the computational complexity of the operation (A5) is notably reduced when applying this substitution and the FFT algorithm is used. Specifically, the computational complexity is reduced from $O(n^{2})$ to O(nlogn).

We here study the convergence speed of the sequence $\left\{\frac{\mathrm{MSE}(W_{C})}{n}-\frac{\mathrm{MMSE}}{n}\right\}$ (i.e., how fast this sequence tends to zero) by assuming that *Y* and *Z* are trigonometric polynomials. Applying [13], p. 11, and Lemma 5, we conclude that there exists K∈[0,∞) such that:

$$\begin{array}{cc} 0\;\leq \;\frac{\mathrm{MSE}(W_{C})}{n}\;-\;\frac{\mathrm{MMSE}}{n}\; & \leq \;\frac{\sqrt{M}\sigma_{1}\left(Z\right)}{\inf Y}\;\left(\;1\;+\;\frac{\sup Y}{\inf Y}\;\right)\;\left(\;\frac{∥{\hat{C}}_{n}\left(Z\right)\;-\;T_{n}{\left(Z\right)∥}_{F}}{\sqrt{n}}\;+\;\frac{\sigma_{1}\left(Z\right)}{\inf Y}\frac{∥{\hat{C}}_{n}\left(Y\right)\;-\;T_{n}{\left(Y\right)∥}_{F}}{\sqrt{n}}\;\right)\\ \; & \leq \;\frac{K}{\sqrt{n}}\;\forall n\in N, \end{array}$$

where $\sigma_{1}\left(Z\right)=\sup_{\omega \in [0,2\pi ]}{∥Z\left(\omega \right)∥}_{2}$ and $\sup Y=\max_{\omega \in [0,2\pi ]}\lambda_{1}\left(Y\left(\omega \right)\right)$. Therefore,

(A6) $$\frac{\mathrm{MSE}(W_{C})}{n}\;-\;\frac{\mathrm{MMSE}}{n}=O\left(\frac{1}{\sqrt{n}}\right).$$

Equation (A6) was proven in [2] for the case M=N=1.

## References

[1] Kolmogorov A.N. (1956). On the Shannon theory of information transmission in the case of continuous signals. IRE Trans. Inf. Theory.

[2] Pearl J. (1973). On coding and filtering stationary signals by discrete Fourier transforms. IEEE Trans. Inf. Theory.

[3] Gutiérrez-Gutiérrez J., Zárraga-Rodríguez M., Insausti X. (2017). Upper bounds for the rate distortion function of finite-length data blocks of Gaussian WSS sources. Entropy.

[4] Zárraga-Rodríguez M., Gutiérrez-Gutiérrez J., Insausti X. (2019). A low-complexity and asymptotically optimal coding strategy for Gaussian vectors sources. Entropy.

[5] Gray R.M. (2006). Toeplitz and circulant matrices: A review. Found. Trends Commun. Inf. Theory.

[6] Gutiérrez-Gutiérrez J., Crespo P.M. (2011). Block Toeplitz matrices: Asymptotic results and applications. Found. Trends Commun. Inf. Theory.

[7] Gutiérrez-Gutiérrez J. (2019). A modified version of the Pisarenko method to estimate the power spectral density of any asymptotically wide sense stationary vector process. Appl. Math. Comput..

[8] Gutiérrez-Gutiérrez J., Zárraga-Rodríguez M., Villar-Rosety F.M., Insausti X. (2018). Rate-distortion function upper bounds for Gaussian vectors and their applications in coding AR sources. Entropy.

[9] Gutiérrez-Gutiérrez J., Iglesias I., Podhorski A. (2011). Geometric MMSE for one-sided and two-sided vector linear predictors: From the finite-length case to the infinite-length case. Signal Process..

[10] Reinsel G.C. (1993). Elements of Multivariate Time Series Analysis.

[11] Rudin W. (1976). Principles of Mathematical Analysis.

[12] Gutiérrez-Gutiérrez J., Crespo P.M. (2011). Asymptotically equivalent sequences of matrices and multivariate ARMA processes. IEEE Trans. Inf. Theory.

[13] Gutiérrez-Gutiérrez J., Zárraga-Rodríguez M., Insausti X., Hogstad B.O. (2017). On the complexity reduction of coding WSS vector processes by using a sequence of block circulant matrices. Entropy.

